# Supplementary material for: Is “Esterhazy II”, an Old Walnut Variety in the Hungarian Gene Bank, the Original Genotype?
Source: Plants (Basel). 2021 Apr 23;10(5):854. doi: 10.3390/plants10050854 (PMC8146819; doi:10.3390/plants10050854)
Supplement: Supplementary file 1 [file plants-10-00854-s001.zip › plants-1152834-supplementary.pdf]

Supplementary

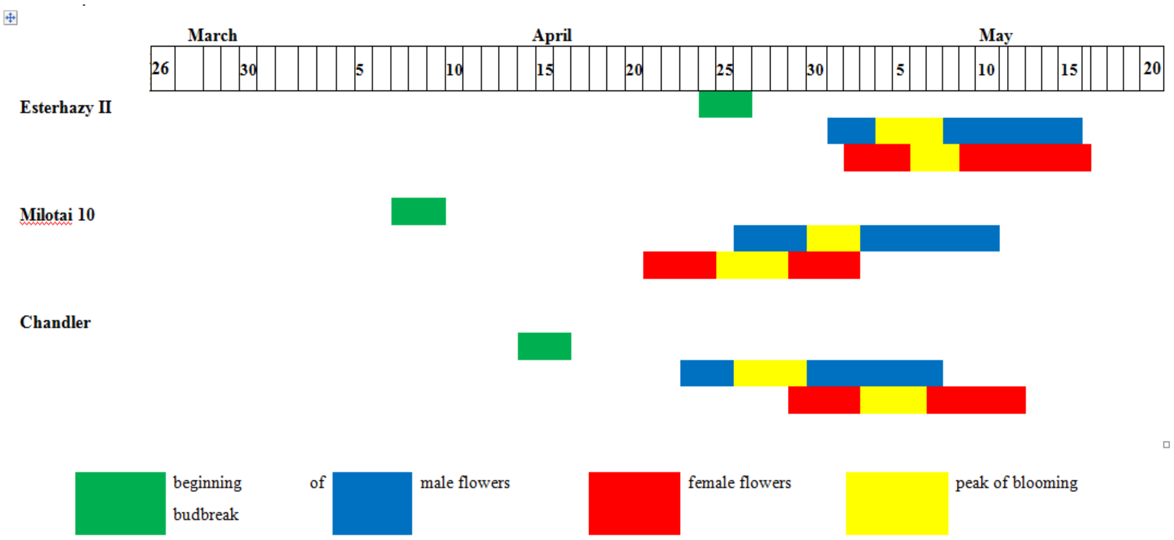

Figure S1. related to Table 2. Phenogram of budbreak, pistillate receptivity and pollen shedding period of Esterhazy II in comparison with Milotai 10 and Chandler (2010-2019).

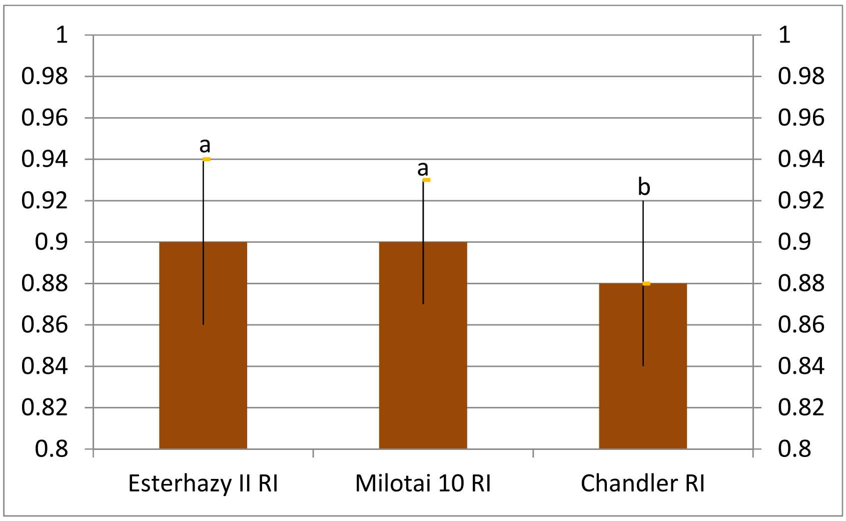

Figure S2. Roundness index (RI) of the 'Esterhazy II', 'Milotai 10', and 'Chandler' nuts (2010-2019) ($SD_{5\%}$= 0.015).

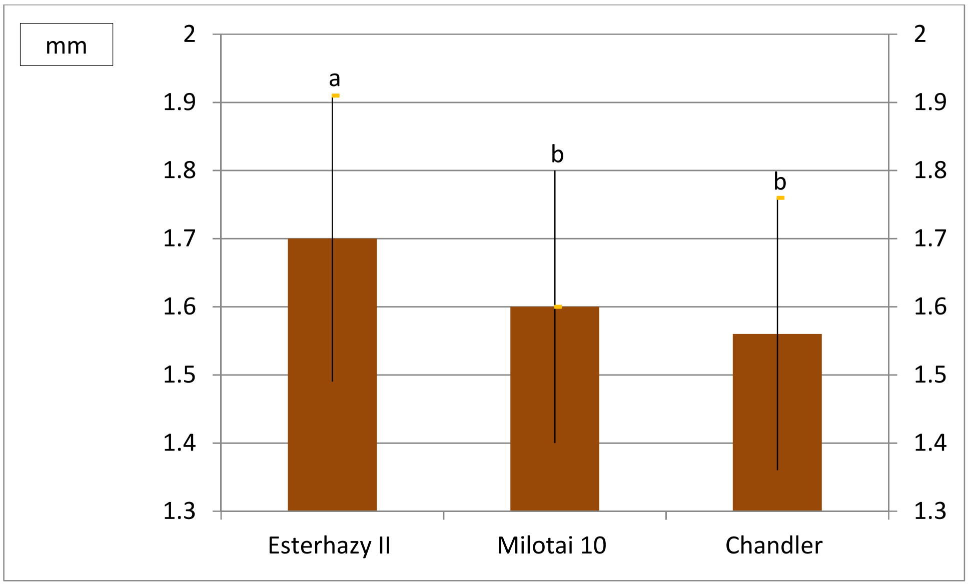

Figure S3. Shell thickness of Esterhazy II, Milotai 10 and Chandler (2010-2019) ($SD_{5\%}$= 0.07).

Table S1. (Related to Figure 5.) Genetic diversity indices of the analysed 18 Juglans regia cultivars applying 8 SSR markers (where N: number of samples, N_a: number of alleles, N_e: effective number of alleles, I: Shannon's information index, H_o: observed heterozygosity, H_e: expected heterozygosity, F: fixation index).

SSR Marker	N	N_a	N_e	I	H_o	H_e	F
**WGA27**	18	2	1.946	0.679	0.389	0.486	0.200
**WGA72**	18	2	1.314	0.403	0.278	0.239	-0.161
**WGA89**	18	4	2.090	0.939	0.611	0.522	-0.172
**WGA118**	18	6	2.919	1.290	0.778	0.657	-0.183
**WGA202**	18	9	4.101	1.703	0.667	0.756	0.118
**WGA276**	18	7	4.154	1.618	0.611	0.759	0.195
**JR 6160**	18	3	1.573	0.655	0.333	0.364	0.085
**JR 1817**	18	3	1.674	0.721	0.278	0.403	0.310
**Min**	18	2	1.314	0.403	0.278	0.239	-0.183
**Max**	18	9	4.154	0.721	0.778	0.759	0.310
**Mean**	18.000	4.500	2.471	1.001	0.493	0.523	0.049
**SE**	0.000	0.906	0.398	0.170	0.069	0.067	0.069
